# Macrophage-Derived Biomarkers of Idiopathic Pulmonary Fibrosis

**DOI:** 10.1155/2011/717130

**Published:** 2010-11-29

**Authors:** E. Bargagli, A. Prasse, C. Olivieri, J. Muller-Quernheim, P. Rottoli

**Affiliations:** ^1^Respiratory Diseases Section, Siena University, 53100 Siena, Italy; ^2^Department of Pneumology, Freiburg University, Germany

## Abstract

Idiopathic pulmonary fibrosis (IPF) is a severe, rapidly progressive diffuse lung disease. Several pathogenetic mechanisms have been hypothesized on the basis of the fibrotic lung damage occurring in this disease, and a potential profibrotic role of activated alveolar macrophages and their mediators in the pathogenesis of IPF was recently documented. This paper focuses on recent literature on potential biomarkers of IPF derived from activated alveolar macrophages. Biomarker discovery and clinical application are a recent topic of interest in the field of interstitial lung diseases (ILDs). Cytokines, CC-chemokines, and other macrophage-produced mediators are the most promising prognostic biomarkers. Many molecules have been proposed in the literature as potential biomarker of IPF; however, a rigorous validation is needed to confirm their clinical utility.

## 1. Introduction

Interstitial lung diseases (ILDs) are a heterogeneous group of rare diseases with different etiopathogenesis and clinical evolution [1]. They include idiopathic pulmonary fibrosis (IPF), a chronic progressive lung disease of unknown etiology, and a prognosis of 3–5 years [2]. The difficulty of early diagnosis of IPF, of differentiating IPF/UIP from various idiopathic interstitial pneumonias and the impossibility of predicting patient outcome (as there exist different phenotypes of IPF) have prompted research into biomarkers [3]. The need for diagnostic and prognostic biomarkers is a topical subject for all chest physicians involved in the management of ILD patients and particularly IPF. Useful biomarkers have to be readily detectable in biological fluids by noninvasive and reproducible procedures and must be demonstrated sufficiently sensitive and specific by appropriate statistical analysis [4]. Identification of new biomarkers of ILD is a growing field of research, favoured by the development of new technologies such as genomics and proteomics that can reveal genetic mutations, polymorphisms, proteins, peptides, and other molecules with a potential role as biological indicators [5, 6]. Modern clinical management of patients with IPF envisages biomarkers with diagnostic and prognostic value, though not a single biomarker, has yet provided sufficient evidence to be implemented in routine patient management [3].

Several pathogenetic mechanisms have been postulated on the basis of fibrotic lung damage occurring in patients with IPF. The inflammatory theory has been partially replaced by the concept of aberrant wound healing due to interactions between epithelial cells and fibroblasts, determining uncontrolled chronic fibroproliferation [7]. More recently, studies on cytokine and chemokine expression in serum and BAL have suggested a potential profibrotic role of activated alveolar macrophages and their mediators in the pathogenesis of IPF [8, 9]. For example, IL13 and some CC-chemokines are directly implicated as mediators of these macrophages [10]. Alveolar macrophages are a heterogeneous population of cells derived from monocytes, with multiple immunological functions. They defend the lungs by phagocytic activity, taking part in aspecific mechanisms of defence as well as specific immune responses via secretory activity [11, 12]. Alveolar macrophages are the most abundant cell population in bronchoalveolar lavage (BAL). Activated alveolar macrophages can release cytokines, growth factors, extracellular matrix proteins, and tissue inhibitors of metalloproteases, contributing to alveolar injury and aberrant lung repair occurring in IPF [13–16]. Study of the macrophage model of activation suggests the existence of classical and alternative modes of activation. The classical mode is mediated by type 1 proinflammatory cytokines, while type 2 cytokines induce the alternative mode, facilitating release of profibrotic mediators and promoting fibrogenetic processes with aberrant production and deposition of collagen in the lungs [15, 16]. Studies on BAL fluid show that alveolar macrophages have a crucial role in the pathogenesis of ILD in general, for example, in sarcoidosis, where they mediate abnormal lymphocyte proliferation and granuloma formation by inducing antigen presentation [17, 18]. In IPF, alveolar macrophages play a profibrotic role through release of fibronectin, insulin growth factor, PDGF, and other mediators [19, 20]. Alternative activated macrophages (M2 phenotype) are crucial in the pathogenesis of IPF enhancing fibrogenesis of fibroblasts by providing profibrogenic factors [21], favouring cell growth, collagen formation, and tissue repair [22]. Prasse et al. demonstrated that alternative activated macrophages trigger a vicious circle between alveolar macrophages and fibroblasts by releasing IL10, IL1 receptor antagonist, and CCL18 (also named PARC or MIP-4), which promote collagen deposition and fibrotic progression of IPF [9]. Moreover, very recently Pechkovsky et al. confirmed the crucial role for M2 macrophages in the pathogenesis of IPF reporting elevated spontaneous production of the chemokines CCL17, CCL18, and CCL22 and increased expression of CD206 by alveolar macrophages from patients with lung fibrosis. They also obeserved that IL-4 was the most powerful inducer of M2-phenotype in alveolar and monocytes from IPF patients [15].

Analysis of recent literature on biomarkers derived from activated alveolar macrophages in IPF is the main topic of this paper.

## 2. Macrophage-Derived Biomarkers of IPF

In the last few years, molecules of different origin have been proposed as potential biomarkers of IPF. Fibrocytes, neutrophils, and alveolar epithelial cells are sources of mediators involved in the pathogenesis of IPF and proposed as candidate biomarkers, for example, KL6, surfactant proteins A and D, or circulating fibrocytes [23–26]. Although many molecules expressed by alveolar macrophages (cytokines such as IL13 and TGF-beta, CC chemokines, and many enzymes, receptors, and growth factors, such as PDGF) have been studied in different biological fluids of IPF patients in order to evaluate their contribution to pathogenesis, only a limited number have been investigated for clinical biomarker potential by seeking correlations between their concentrations and parameters of clinical prognostic [3, 15, 21, 27–32].

**CC Chemokine Ligand 18 (CCL18)**

has been widely studied in BAL, serum, and tissue of IPF patients, proving a very promising biomarker derived from alternatively activated alveolar macrophages (Th2 cytokines and IL10 induce its biosynthesis) and highly expressed in the lungs [15, 33–35] (Table 1). In 2006, a group of German researchers demonstrated that normal human alveolar macrophages express CCL18 spontaneously, but its production is much higher (more than 100-fold) in IPF patients than controls [15]. They also demonstrated that contact and exposure of fibroblasts to native collagen induces spontaneous release of CCL18 by M2 macrophages, which also upregulate collagen production by lung fibroblasts [15]. CCL18 has therefore been recognised as a mediator of positive feedback between alveolar macrophages and fibroblasts, promoting collagen deposition in IPF. The effectiveness of CCL18 as a biomarker of IPF activity was later documented by measuring its concentrations in serum, BAL, and BAL cell supernatants from a group of IPF patients [33]. Concentrations in serum strictly reflected the time trend of certain functional parameters (such as TLC) in these patients. An inverse correlation was also found between spontaneous release of BAL CCL18 and TLC or DLCO; as well, a direct correlation was demonstrated between BAL CCL18 concentrations and BAL neutrophil and eosinophil counts [33]. The prognostic value of CCL18 was recently reported in a prospective study that documented significantly higher mortality in IPF patients with serum CCL18 concentrations above 150 ng/ml [35]. High levels of CCL18 in serum were associated with high risk of disease progression. CCL18 is therefore an important M2 biomarker; its concentrations are elevated not only in serum of IPF patients but also in other ILDs with active fibrosis, and to a lesser extent in serum of patients with bronchial asthma and rheumatoid arthritis [33, 34]. Extension of CCL18 studies to a larger population will enable definition of its potential use in clinical practice for the management of IPF patients. This chemokine could be particularly useful, especially as a prognostic indicator of patient survival.

Alveolar macrophages can release other CC chemokines that could be implicated in fibrogenesis, including *CC chemokine ligand 2* (CCL2 or monocyte chemoattractant protein-1, MCP-1) that together with CCL3 and CCL4, proved higher in BAL of IPF patients than controls [8, 32, 36–38]. CCL2 levels in BAL were inversely correlated with DLCO values and arterial oxygen tension in IPF [32, 36]. Shinoda et al. suggested that elevated concentrations of CCL2, CCL17, and CCL22 in BAL fluid may be predictive of poor outcome in patients with IPF, being associated with poor survival rate [32]. Concentrations of CCL-2 can also be increased not only in BAL but also in serum and of IPF patients (Table 1); indeed, Suga et al. suggested that the clinical evolution of the disease is correlated with serum CCL2 chemokine concentrations [37]. The potential application of CCL2 as a clinical biomarker of IPF seems to be limited by its low specificity; its serum concentrations are higher in connective tissue lung diseases than in IPF, and common nonrespiratory diseases such as atherosclerosis may raise levels, acting as a confounding factor [38, 39].

**IL-8**

(also known as neutrophil chemotactic factor) is a major mediator of inflammatory response. Produced by alveolar macrophages, epithelial cells, and other cell types, it functions as a chemoattractant for neutrophilic granulocytes, macrophages, endothelial cells, and mast cells and as a potent angiogenic factor [40]. Concentrations of IL-8 in serum can be influenced by several factors, including infectious diseases (Table 1) [41]. More than 10 years ago, Ziegenhagen et al. reported higher concentrations of this CXC chemokine in serum and BAL of patients with IPF than normal controls [42]. They also reported positive correlations between serum and BAL concentrations of IL-8 and neutrophil percentages in BAL [42]. Serum levels of this chemokine were inversely correlated with lung function data (DLCO, VC, TLC) and oxygen pressure measured by blood gas analysis in this population of IPF patients. The authors suggested that IL-8 may be a useful clinical marker in the followup of IPF patients [42]. After this study, other research confirmed the very high levels of IL8 in serum of IPF patients, as well as inverse correlations with functional parameters (mainly FVC) [43, 44]. A Japanese study indicated that high serum levels of IL-8 can be observed in patients with active disease, while stable IPF patients had low serum concentrations of IL-8 [44]. The correlation between IL8 concentrations and mortality rate has never been tested. IL-8 is a potential biomarker of IPF with limited specificity that could possibly be included in a cluster of biomarkers to define their effective prognostic power in a larger group of patients.

The complex pathogenetic mechanisms and lack of diagnostic and prognostic biomarkers of IPF have driven proteomic research in the last few years. Several proteomic studies have been done, and a proteomic approach has been used to analyse protein composition of BAL from different ILD, including IPF, identifying proteins of interest [6, 45–49]. Calgranulin B and MIF, produced by activated alveolar macrophages were among those expressed differently in IPF patients than controls [49–51] (Table 1).

**Calgranulin B (S100A9 or MRP14)**

**Figure 1:**
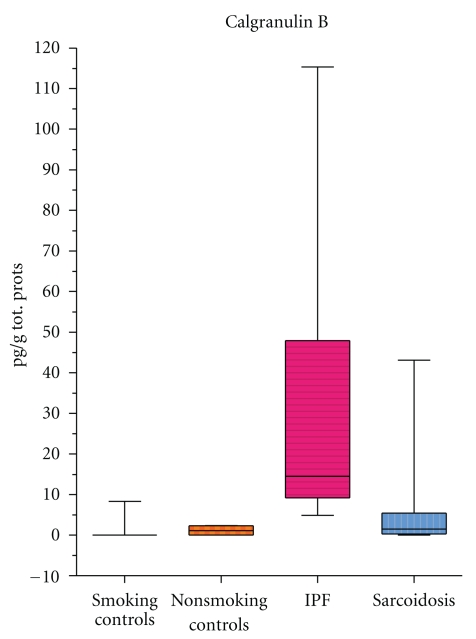
Calgranulin B concentrations in BAL of sarcoidosis, idiopathic pulmonary fibrosis patients and healthy controls (smokers and nonsmokers). The box extends from the 25th percentile to the 75th percentile, with a line at the median (the 50th percentile). Prism plots the whiskers to extend above and below the box to show the highest and lowest values [61].

Is a S100 protein produced by monocyte-macrophages, neutrophils, and other cells mainly in response to chronic inflammatory processes [52]. S100A9 is involved in the regulation of a number of cell processes such as cell cycle progression and differentiation [53]. Recent data suggests that this protein has a crucial role in inflammation, cancer, and fibrotic lung remodeling [54]. It interacts with extracellular matrix proteins, stimulates fibroblast proliferation, and regulates transendothelial migration of leukocytes and adhesion of neutrophils to fibrinogen via beta 2 integrin Mac-1 [55, 56]. Its interactions with RAGE receptors (other profibrotic agents implicated in the regulation of extracellular matrix degradation in IPF) facilitate neutrophil migration from endothelium to interstitium, adhesion of neutrophils to fibronectin, and fibrotic lung remodeling [57]. Calgranulin B is overexpressed in breast, lung and gastrointestinal cancer, autoimmune inflammatory diseases, and COPD and cystic fibrosis [58–60]. In 2002, calgranulin B was identified for the first time in BAL of IPF and sarcoidosis patients by a proteomic approach using gel matching and MALDI-TOF mass fingerprinting [49]. Percentage volumes of calgranulin B proved significantly higher in BAL of IPF patients than sarcoidosis patients or patients with pulmonary fibrosis associated with systemic sclerosis or controls (Figure 1) [45]. Quantitative analysis (ELISA) confirmed the very high concentrations of this protein in BAL of IPF patients, presumably due to the elevated number and massive activation of polymorphonuclear cells in this disease (indeed calgranulin B concentrations in BAL showed a positive correlation with neutrophil/eosinophil percentages in BAL) [50]. The suitability of BAL calgranulin B as a disease biomarker was suggested by an inverse correlation with FVC and DLCO; the highest concentrations of calgranulin B in BAL were observed in patients with the lowest FVC and DLCO values [61]. Elevated BAL concentrations of calgranulin B in IPF patients and a correlation with BAL neutrophil counts were recently also confirmed by Korthagen et al. who additionally reported a moderate increase in this protein, correlated with radiological stages, in BAL of sarcoidosis patients [62]. Further study of calgranulin B as a potential indicator of IPF severity and sarcoidosis is warranted. The protein expression has to be analysed in serum of IPF patients and in order to verify its specificity also in serum and BAL samples of patients with different interstitial lung diseases.

Another interesting protein identified by a proteomic approach in BAL of patients with IPF and differently expressed in patients than controls is macrophage migration inhibitory factor, MIF. MIF is a pleiotropic proinflammatory cytokine produced by activated macrophages and potentially involved in IPF pathogenesis for its numerous functions including regulation of cell redox status, inhibition of apoptosis, induction of matrix metalloproteinases, and regulation of Th1/Th2 immune responses [51]. Its concentrations in BAL of IPF patients are very high and directly correlated with BAL neutrophil percentages. Immunohistochemical analysis of MIF revealed enhanced expression from actively fibrosing areas, such as fibroblast foci and lung remodeling zones [51]. The role of MIF as a disease biomarker needs to be investigated. Potential correlations with clinical parameters (including mortality rate) in IPF patients and the expression of MIF in other interstitial lung diseases have to be furtherly analysed.

## 3. Conclusion

The recent literature on IPF indicates great interest in developing diagnostic and prognostic biomarkers and in defining the potential role of activated macrophages in the pathogenesis of IPF. Potential IPF biomarkers derived from activated alveolar macrophages, currently being studied, include CCL18, CCL2, IL-8, and calgranulin B. 

The discovery and development of biomarkers has to be conducted in a rigorous manner; studies on potential biomarkers have to be performed on a large scale, and appropriate statistical analysis is necessary to validate a molecule as a real biological indicator [4, 63]. In the last few years, only a limited number of molecules analysed in ILD have made the transition from candidate to true biomarker status because of the difficulties associated with validating biomarkers. Among those analysed in this paper, CCL18 seems the most promising macrophage-derived biomarker of IPF, especially because it is clearly correlated with patient mortality [35]. However, before serum assay of CCL18 can be used in the clinical management of IPF patients, further analysis on a larger populations is required. 

Macrophage-derived biomarkers of IPF are proteins overexpressed in serum, BAL samples, or lung tissue of patients with IPF compared with controls. An alternative approach to identify potential bioindicators of IPF has been suggested by Ren et al. who reported evidence of impaired transcription in IPF macrophages. The authors concluded that the detection of a decrease in a specific protein produced by macrophages would be an alternative method to identify potential bioindicators of this severe interstitial lung disease [64].

Sophisticated methods applied to different biological fluids are revealing interesting new groups of molecules that could provide future biomarkers of IPF, useful for fine phenotyping of patients and for clinical management of the disease.

## Figures and Tables

**Table 1:** The main features of macrophage-derived biomarkers of idiopathic pulmonary fibrosis.

	CCL18	IL8	CCL2	S100A9	MIF
Cellular production	Monocytes/ Macrophages, Dentritic cells	Macrophages, Epithelial cells	Monocytes/ Macrophages, Fibroblasts, Epithelial cells, Endothelial cells	Monocytes/ Macrophages, Neutrophils	Macrophages, T cells
Biological fluids	Serum, Lung tissue, BAL	Serum, BAL	Serum, BAL	BAL, Lung tissue	BAL, Lung tissue
Concentrations in IPF versus controls	Increased	Increased	Increased	Increased	Increased
Correlation studies with clinical parameters	YES	YES	YES	YES	None
Studies on other ILDs (specificity)	Sarcoidosis, NSIP, Systemic sclerosis, Hypersensitivity pneumonitis, Vasculitis, Smoking-related ILD	Sarcoidosis, Systemic sclerosis, BOOP, Vasculitis, Hypersensitivity, pneumonitis	Sarcoidosis, Systemic sclerosis Hypersensitivity, pneumonitis, BOOP, AIP, Silicosis	Sarcoidosis, Systemic sclerosis	Sarcoidosis, Wegener's granulomatosis, Systemic sclerosis, Hypersensitivity pneumonitis
Studies on other lung diseases	Malignancies, TB, Asthma	Bronchiolitis, Cancer, Asthma COPD, Cystic fibrosis, ARDS	Malignancies, Asthma, TB	Malignancies, Pneumonia	Lung cancer, Pneumoniae, Asthma, ARDS

## Abbreviations

BAL: Bronchoalveolar lavage
IPF: Idiopathic pulmonary fibrosis
ILD: Interstitial lung diseases
2-DE: Two-dimensional electrophoresis.
